# A machine learning approach to predicting vascular calcification risk of type 2 diabetes: A retrospective study

**DOI:** 10.1002/clc.24264

**Published:** 2024-04-02

**Authors:** Xue Liang, Xinyu Li, Guosheng Li, Bing Wang, Yudan Liu, Dongli Sun, Li Liu, Ran Zhang, Shukun Ji, Wanying Yan, Ruize Yu, Zhengnan Gao, Xuhan Liu

**Affiliations:** ^1^ Department of Endocrinology Dalian Municipal Central Hospital Dalian China; ^2^ Graduate School Dalian Medical University Dalian China; ^3^ Laboratory Pathology Department Ningbo Clinical Pathology Diagnosis Center Ningbo China; ^4^ Department of Neuroendocrine Pharmacology, School of Pharmacy China Medical University Shenyang China; ^5^ International Center, InferVision Beijing China

**Keywords:** cross sectional study, *k*‐nearest neighbor, machine learning, Naive Bayes, prediction model, type 2 diabetes mellitus, vascular calcification

## Abstract

**Background:**

Recently, patients with type 2 diabetes mellitus (T2DM) have experienced a higher incidence and severer degree of vascular calcification (VC), which leads to an increase in the incidence and mortality of vascular complications in patients with T2DM.

**Hypothesis:**

To construct and validate prediction models for the risk of VC in patients with T2DM.

**Methods:**

Twenty‐three baseline demographic and clinical characteristics were extracted from the electronic medical record system. Ten clinical features were screened with least absolute shrinkage and selection operator method and were used to develop prediction models based on eight machine learning (ML) algorithms (*k*‐nearest neighbor [*k*‐NN], light gradient boosting machine, logistic regression [LR], multilayer perception [(MLP], Naive Bayes [NB], random forest [RF], support vector machine [SVM], XGBoost [XGB]). Model performance was evaluated using the area under the receiver operating characteristic curve (AUC), accuracy, and precision.

**Results:**

A total of 1407 and 352 patients were retrospectively collected in the training and test sets, respectively. Among the eight models, the AUC value in the NB model was higher than the other models (NB: 0.753, LGB: 0.719, LR: 0.749, MLP: 0.715, RF: 0.722, SVM: 0.689, XGB:0.707, *p* < .05 for all). The *k*‐NN model achieved the highest sensitivity of 0.75 (95% confidence interval [CI]: 0.633–0.857), the MLP model achieved the highest accuracy of 0.81 (95% CI: 0.767–0.852) and specificity of 0.875 (95% CI: 0.836–0.912).

**Conclusions:**

This study developed a predictive model of VC based on ML and clinical features in type 2 diabetic patients. The NB model is a tool with potential to facilitate clinicians in identifying VC in high‐risk patients.

## Graphical abstract

For the first time ever, our study utilized artificial intelligence to build the prediction model for vascular calcification (VC) risk in Chinese type 2 diabetics aged 27–92. The 10 important characteristics that affect VC were identified and ranked. Serum uric acid, a newly discovered possible factor influencing VC, has attracted our attention.

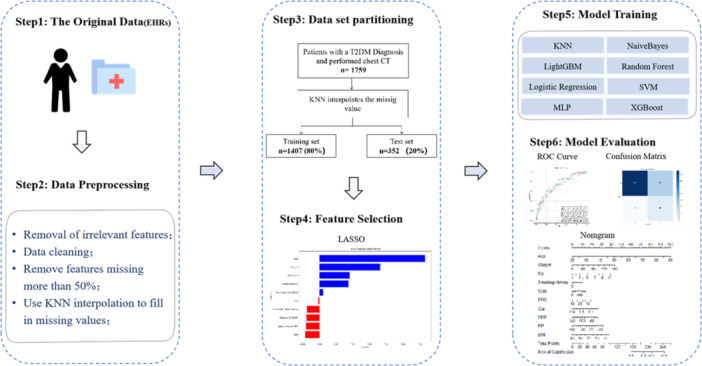

**Abbreviations**
AUC: the area under the receiver operating characteristic curve
CACS: coronary artery calcification score
CVD: cardiovascular disease
*k*‐NN: *k*‐nearest neighbor
LightGBM: light gradient boosting machine
LR: logistic regression
MACE: major adverse cardiac events
ML: machine learning
MLP: multilayer perceptron
NB: Naive Bayes
non‐VC: nonvascular calcification
RF: random forest
SVM: support vector machine
T2DM: type 2 diabetes mellitus
VC: vascular calcification
XGB: XGBoost

## 1 INTRODUCTION

Vascular calcification (VC) is ectopic calcification caused by the disorder of calcium and phosphorus metabolism, which is a highly complex, active, and multidirectional active process, leading to increased stiffness of the vessel wall, decreased compliance, thrombosis, and plaque rupture.^1^, ^2^, ^3^ In recent years, the prevalence of VC has increased, with high morbidity and mortality, and has been shown to be an independent and strong predictor of cardiovascular disease (CVD) and major adverse cardiac events (MACE).^4^, ^5^ As the mechanism of VC has not been clarified, there are no effective therapeutic drugs or methods to prevent or reverse VC,^1^, ^6^ so early identification of high‐risk groups of VC has clinical significance. Besides the prevalence of VC in patients with diabetes is much higher than those without diabetes^7^, ^8^, ^9^, ^10^, ^11^ and the prevalence of diabetes mellitus (DM) is gradually increasing, making it more likely to develop CVDs in the future.^12^, ^13^

The density and extent of calcified plaques in the intima of large arteries, which are part of atherosclerotic plaques, can be assessed on contrast‐free computed tomography (CT) of the chest.^12^, ^14^, ^15^, ^16^ But the traditional diagnosis of VC by radiologist report is time‐consuming and easy to miss and given the wide variety of conditions under which VC occurs, as well as the technical limitations of clinical calcification imaging, estimating the total social cost of VC is difficult and there is an urgent need to address the management of this disease.^3^ However with the tremendous computational power, machine learning (ML) has recently been employed in a variety of medical problem solving or outcome predictions because it shows greater accuracy compared to conventional statistical methods.^17^, ^18^, ^19^ In recent years, predictive models based on ML techniques to predict different diseases have been widely used. It is a process of extracting knowledge from data and finding potential connections.^20^, ^21^ Previous studies have found to automatically predict coronary artery calcification score (CACS) using chest CT, based on deep learning algorithms, which has already shown promising results in image deep learning tasks.^22^, ^23^ However, as far as we know, there is no risk prediction model based on ML algorithms to predict the VC using only clinical variables in a type 2 diabetic population. To identify patients at risk for VC and to improve disease characteristics and solve a problem that needs to be further addressed in the field of artificial intelligence, the aim of this study was to establish a risk prediction model based on ML algorithms to predict the VC using only clinical variables in a type 2 diabetic population.

In this study, electronic medical record retrieval system was used to select patients with type 2 diabetes who were admitted to the Department of Endocrinology of Dalian Medical University from January 1, 2010, to January 1, 2021. The prediction model of VC risk is established based on ML algorithm. This study also explored the important characteristics affecting the occurrence of VC, so as to provide a basis for further research on the prevention and management of VC.

## 2 MATERIALS AND METHODS

### 2.1 Study population

Retrospective analysis was performed on the data of inpatients in the Department of Endocrinology of Dalian Medical University from January 1, 2010, to January 1, 2021, extracted by electronic medical record system.

Inclusion criteria: (1) Age ≥ 18 years with the diagnosis of type 2 diabetes; (2) at least performed one chest CT scan. Exclusion criteria: (1) pregnant or lactating patients; (2) neurological diseases (e.g., brain trauma or epilepsy); severe mental illness (e.g., schizophrenia); (3) factors that may lead to secondary osteoporosis, such as chronic liver and kidney diseases, after subtotal gastrectomy, and so on; (4) those who have recently taken drugs that inhibit uric acid production, promote uric acid excretion, diuretics and other drugs that affect uric acid metabolism; (5) patients suffering from malignant tumors or hereditary diseases; (6) patients with acute infection and acute complications; (7) patients with severe heart failure, liver failure and renal failure; (8) parathyroid dysfunction and other diseases affecting calcium and phosphorus metabolism; (9) missing clinical information.

This study was a retrospective analysis, and the required data were the clinical routine data of hospitalized patients. All patients had signed the pan‐informed consent at admission, which was approved by the ethics committee of Dalian Central Hospital, and it was unnecessary to sign the informed consent again.

### 2.2 Data source

**Figure 1:**
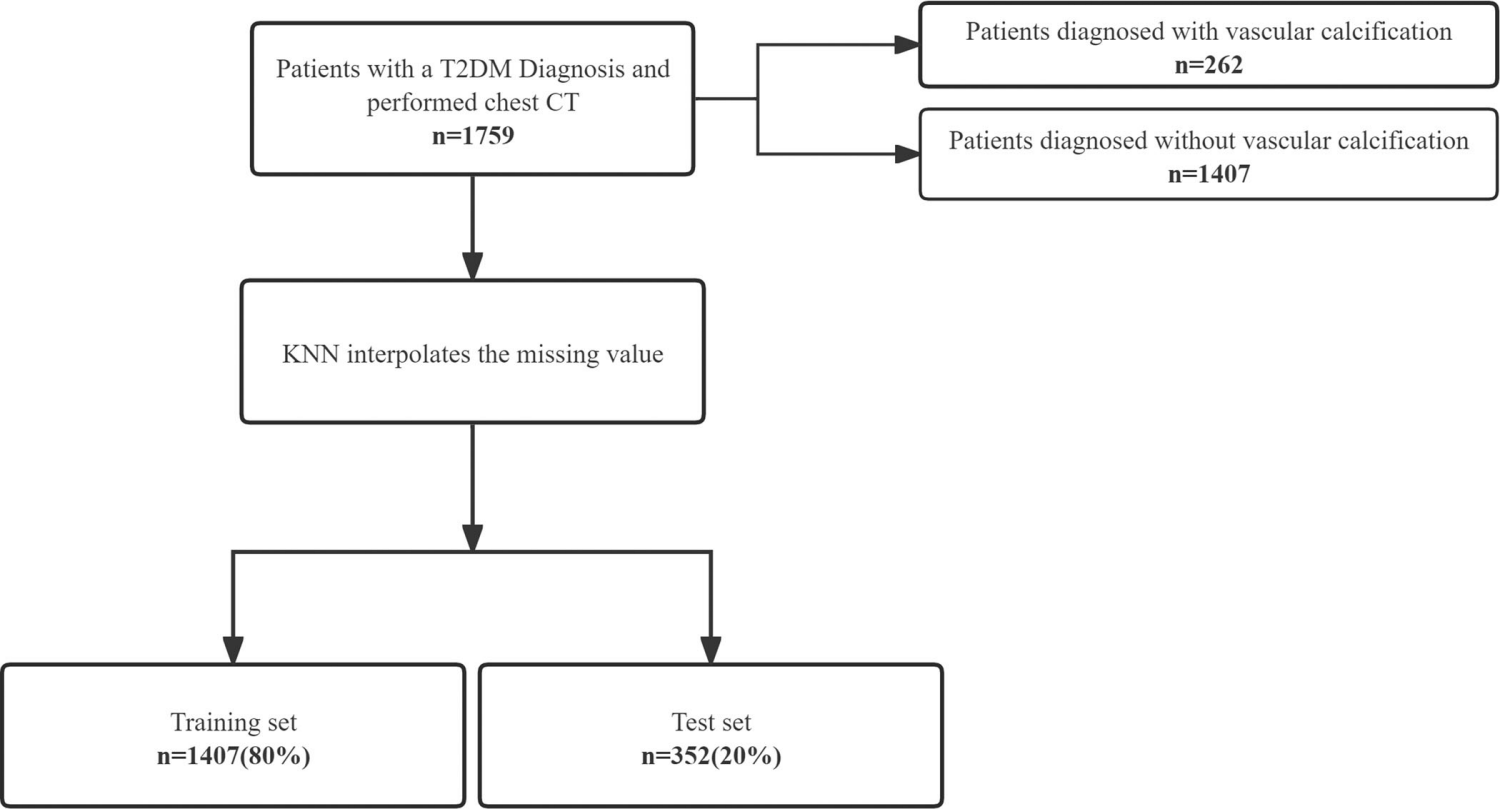
Flow diagram of patient selection. CT, computed tomography; *k*‐NN, *k*‐nearest neighbor; T2DM, type 2 diabetes mellitus.

With the criteria mentioned above, 1759 patients were selected for this study, including 262 patients diagnosed with VC at baseline and 1497 ones without VC. Patients were randomly split 4:1 into a training set (*n* = 1407) and a test set (n = 352) (Figure 1).

### 2.3 Diagnostic criteria

Diagnostic criteria of T2DM are according to the Guidelines for Prevention and Treatment of Type 2 Diabetes in China (2020). VC was defined as the diagnosis of large vessel calcification in the mediastinal window (the presence of “calcification spot” and “calcification spot” in the aorta or coronary artery with CT value greater than 130) on chest CT.^21^ Chest CT images were confirmed by at least two attending radiologists.

### 2.4 Clinical variables

The information of patients on demographics, medical history, medication, and laboratory data was extracted from the electronic medical record system at baseline (Supporting Information S1: Table S1). These variables are commonly associated with the risk of VC, including nine categorical variables and 14 continuous variables (Supporting Information S1: Table S1). The smoking status was defined as: (1) smoking: patients who smoked more than 100 cigarettes on the index date and did not quit smoking; (2) nonsmokers: patients who have never smoked or who have smoked before but quit smoking 30 days before the index date. The drinking status was defined as: (1) drinking: drinking and abstinence less than 1 year; (2) nondrinkers: never drank alcohol or abstinence for at least 1 year.

Hypertension was defined as a systolic blood pressure of ≥140 mmHg and/or a‐diastolic blood pressure ≥ 90 mmHg, measured twice or more on different days, or patients have been previously diagnosed with hypertension or patients are currently taking antihypertensive medication.^27^ Blood samples were collected the next morning after hospital admission with at least 12‐h fasting. Hemoglobin A1c was measured using the glycosylated hemoglobin analyzer (TOSOH company of Japan, HLC‐723G8). Four items of blood lipids (high‐density lipoprotein cholesterol, total cholesterol, low‐density lipoprotein cholesterol, and triglyceride), alanine transaminase, aspartate transaminase, gammaglutamyl transpeptidase, serum creatinine, serum uric acid (SUA), and fasting plasma glucose (FPG) were detected by automatic biochemical analyzer (ADVIA2400 biochemical system; Siemens). Body mass index (BMI) was calculated as weight (kg) divided by the square of height in meters (m^2^); Blood pressure was measured on the right arm in sitting position three times consecutively at 5‐min intervals, with the mean of the three measurements used for analysis.

### 2.5 Data preprocessing and feature selection techniques

**Figure 2:**
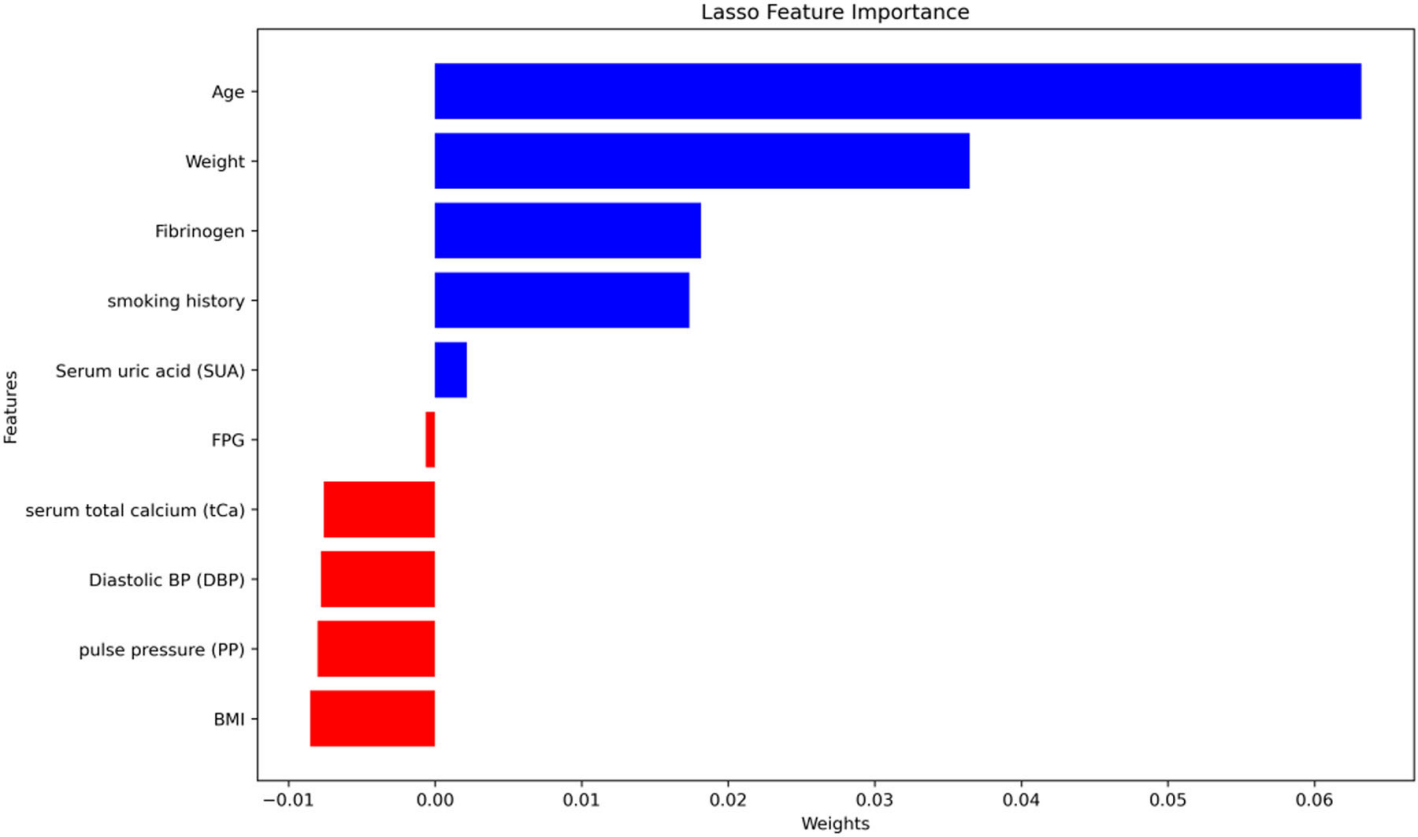
Feature importance ranking. BMI, body mass index; FPG, fasting plasma glucose.

We initially collected 294 clinical features from electronic medical record system. After removing irrelavent features and features with a missing rate over 30%, 23 features remained (Supporting Information S1: Table S1). Features with missing values were first imputed with *k*‐nearest neighbors technique (*k*‐NN Imputer). This method fill the missing value with existing value from other patients which has the smallest Euclidean Distance to it. The distance is calculated with features that neither is missing. We set n_neighbors=1 such that both categorical variables and continuous variables could be imputed. After applying standardization to all features, least absolute shrinkage and selection operator (LASSO) feature selection technique was employed to reduce redundant variables and decrease overfitting. Among the 23 clinical variables, 10 variables were kept after feature selection, and their feature importance was plotted in Figure 2. For reason that the number of positive and negative patients was extremely imbalanced, we applied synthetic minority oversampling technique to balance our training data and the ratio between positive and negative samples after oversampling was 7:10.

### 2.6 ML models construction

In this study, eight ML models were constructed. Clinical and laboratory features were screened with LASSO method and were used as inputs for the eight ML algorithms to predict VC in type 2 diabetes. ML models containing the random forest (RF), support vector machine (SVM), *k*‐NN, light gradient boosting machine (LightGBM), LR, multilayer perception (MLP), NB, and XGBoost (XGB) were used to assess the risk of the VC.

Internal validation of the model and optimization of hyperparameters: the method of 10‐fold cross validation is adopted on the training set. On the training set, the data set is randomly divided into 10 parts with nearly equal number of samples, and nine parts are used as training data and the remaining one part is used as a test to obtain the corresponding accuracy rate (or error rate) of each test. By averaging the measurements of multiple tests, the better evaluation of the model performance is guaranteed, so as to further train and internally verify the prediction model. At the same time, the method of Grid Search CV is used to find the parameters with the highest accuracy from all parameters and optimize the optimal hyperparameters of the model. The best model parameters were showed in Supporting Information S1: Table S2.

### 2.7 Evaluation method and analysis index

Performances of models were evaluated by the area under the receiver operating characteristic (ROC) curve (AUC), prediction accuracy, sensitivity, and specificity. Finally, the study explore the best VC risk prediction model. In this study, the important features screened by LASSO regression method were included to further construct the nomogram of the occurrence risk of VC in diabetic patients, visualize the results of LR model, and obtain the occurrence probability of VC in each patient through the conversion function between the total score and the probability of outcome, which is beneficial to early detection of high‐risk groups of the occurrence risk of VC (Supporting Information S1: Figure S1).

### 2.8 Statistical analysis

All statistical analyses were performed by using R statistical and computing software version 4.2.1. *χ* ^2^ test was used for categorical variables, and two sample *t* test for continuous variables. Two‐tailed hypothesis testing was used. *p* < .05 indicated a statistically significant result. LASSO features were used to identify clinical features associated with high risk of VC. The sklearn library of Python 3.6 software was used to establish ML models, and the sensitivity, specificity, accuracy, and AUC were used to evaluate the efficiency of each model.

## 3 RESULTS

### 3.1 Baseline characteristics of the study population

A total of 1759 patients with type 2 diabetes were included in this analysis. Patients had a median age of 64.75 years, and 830 (47.2%) patients were female. Patients were randomly split into training (1407) and test sets (352). Baseline demographic and clinical characteristics of patients with or without VC are shown in Supporting Information S1: Table S3.

By comparing the features of patients in the training set and the test set, significant differences were found in the distribution of fibrinogen (*p* < .05). it found that expect fibrinogen level, there was no significant difference (*p* > .05) in age, gender, smoking history, diastolic blood pressure, pulse pressure, body mass index, fasting glucose, and so on (Supporting Information S1: Table S3).

### 3.2 Model performance

**Figure 3:**
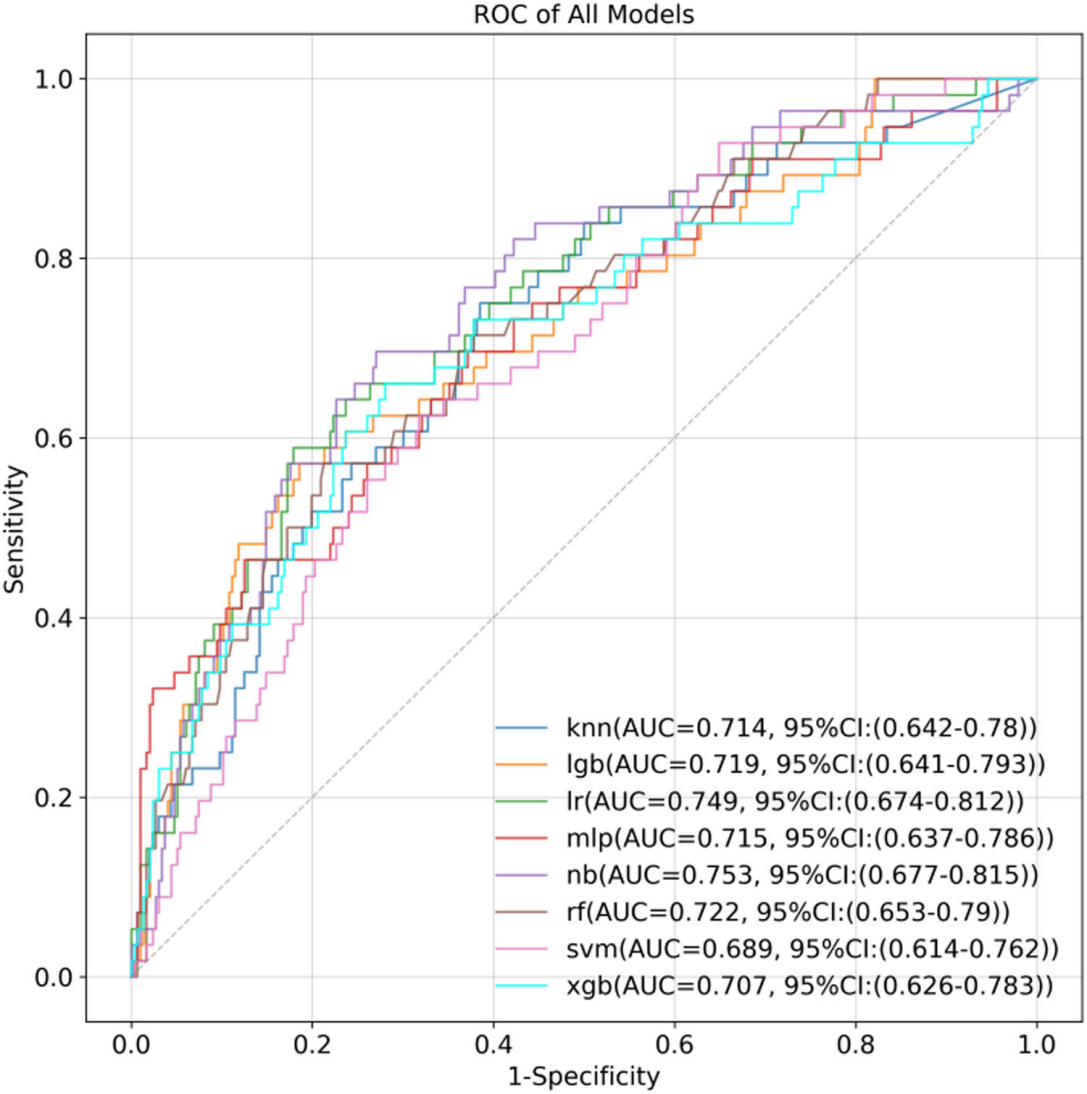
Receiver operating characteristics (ROC) curves of the ML models. Results of AUCs showing the performance of the different ML algorithms to predict VC, respectively. AUC, area under the curve; *k*‐NN, *K*‐nearest neighbor; LightGBM, light gradient boosting machine; LR, logistic regression; ML, machine learning; MLP, multilayer perceptron; NB, Naive Bayes; RF, random forest; SVM, support vector machine; VC, vascular calcification; XGBoost, extreme gradient boosting.

The 10 selected variables were used as inputs for the eight ML algorithms to predict VC in T2DM. Performance evaluation of the models generated by the eight ML algorithms is shown in Table 1. According to the experimental results, the NB model had the highest AUC (0.753, 95% CI: 0.677–0.815) on the test sets (*N* = 352), the ROC curves are presented in Figure 3. In the prediction of VC in type 2 diabetic patients (non‐VC = 296, VC = 56), 216 cases of non‐VC were correctly predicted, and 39 cases of VC were correctly predicted. The Confusion Matrix of the NB model and common performance metrics were calculated (Supporting Information S1: Figure S2). The best accuracy achieved for predicting the diabetic VC was 0.810, (95% CI: 0.767–0.852) (MLP model).

**Table 1:** The performance metrics of the cross‐validated machine learning algorithms on the test data.

Model	AUC (95% CI)	Sensitivity (95% CI)	Specificity (95% CI)	Accuracy (95% CI)
*k*‐NN	0.714 (0.642–0.78)	**0.750** (0.633–0.857)	0.615 (0.561–0.67)	0.636 (0.588–0.685)
LGB	0.719 (0.641–0.793)	0.571 (0.45–0.707)	0.814 (0.769–0.858)	0.776 (0.73–0.821)
LR	0.749 (0.674–0.812)	0.589 (0.453–0.717)	0.821 (0.775–0.866)	0.784 (0.741–0.827)
MLP	0.715 (0.637–0.786)	0.464 (0.333–0.588)	**0.875** (0.836–0.912)	**0.810** (0.767–0.852)
NB	**0.753** (0.677–0.815)	0.696 (0.567–0.812)	0.73 (0.679–0.78)	0.724 (0.676–0.767)
RF	0.722 (0.653–0.79)	0.571 (0.45–0.705)	0.787 (0.744–0.832)	0.753 (0.71–0.798)
SVM	0.689 (0.614–0.762)	0.643 (0.509–0.766)	0.655 (0.599–0.71)	0.653 (0.602–0.705)
XGB	0.707 (0.626–0.783)	0.661 (0.541–0.78)	0.72 (0.668–0.771)	0.71 (0.662–0.756)

*Note*: Model performance was evaluated using the AUC, accuracy, and precision.

Abbreviations: AUC, area under the receiver operating characteristic curve; CI, confidence interval; *k*‐NN, *k*‐nearest neighbor; LightGBM, light gradient boosting machine; LR, logistic regression; MLP, multilayer perceptron; NB, Naive Bayes; RF, random forest; SVM, support vector machine; XGB, XGBoost.

### 3.3 Development of a nomogram

LASSO feature selection showed that age, weight, fib, smoking history, SUA, FPG, serum total calcium, diastolic blood pressure, pulse pressure, and BMI were clinically important features of VC in T2DM (Figure 2). We selected 10 clinical features to construct nomogram to predict the risk of VC (Supporting Information S1: Figure S1).

## 4 DISCUSSION

This study is the first to apply ML approaches to VC risk prediction in Chinese patients with type 2 diabetes, and the model includes personal risk factors to identify high‐risk patients. With the high flexibility of ML and its excellent ability to deal with the relationship between complex features and results, the area under the surface of the VC risk prediction model in this study was 0.753 (95% CI: 0.677–0.815) with good prediction accuracy. In this study, we used electronic health record data to construct eight ML‐based models for the risk of VC in patients with type 2 diabetes, and three of them showed good performance. The NB model had the highest AUC, which was better than the other models (0.753; 95% CI: 0.677–0.815), *k*‐NN model had the highest sensitivity (0.75), MLP model had the highest specificity (0.875), and the highest accuracy (0.81). In the ML model of VC, the NB model (AUC = 0.753; 95% CI: 0.677–0.815), *k*‐NN model had the highest sensitivity (0.75), MLP model had the highest specificity (0.875), and the highest accuracy (0.81). This indicates that after inputting features, our ML model can predict VC risk in patients with type 2 diabetes. To determine the key features affecting the prediagnosis of VC, LASSO algorithm was used to obtain the importance ranking of features, and a total of 10 features were obtained: age at diagnosis, admission weight, fibrinogen, smoking history, body mass index, admission pulse pressure, diastolic blood pressure, total blood calcium, blood uric acid, and fasting blood glucose. Based on LR model, this study established a nomogram of the occurrence of VC in type 2 diabetes patients to realize LR visualization and easy operation, and to assess the individual risk of VC at any time. In clinical application, patients or physicians can input individual clinical characteristics into the nomogram at any time and anywhere. The sum of individual scores of these 10 predictive variables can be obtained on the probability line to obtain the corresponding scale, that is, the probability of occurrence of VC, so as to evaluate the risk of VC in patients with type 2 DM.

In previous studies on the prediction of VC based on ML, a prediction model for VC of chronic kidney disease was established.^25^ Four ML models (LR, RF, SVM, *k*‐NN) were analyzed, and the performance of LR model in ML was the best. However, the target population of this model is mainly chronic kidney disease population, and there is no influence of glycemic factors in the course of disease progression, so it cannot be directly used to predict VC in diabetic population. In another study, Park et al.^28^ built a prediction model for VC in the general population of South Korea in 2021. The target population of that study was community residents. Basic studies showed that the pathophysiological process of their VC in this study was not completely consistent with that in diabetic population, nor was it applicable to the prediction of VC in patients with T2DM. This study is the first one to establish a prediction model of VC based on ML in type 2 diabetes population in China, which can provide early assessment and intervention for type 2 diabetes population prone to VC, guide clinical practice, and provide support for clinical evaluation of VC and to prepare for drug development such as VC inhibitors.

In recent years, the prevalence of VC has increased, with high morbidity and mortality, especially in diabetic patients.^12^, ^13^ In addition, VC has been identified as a complication of diabetes, as shown in preclinical mouse models of arterial calcification.^9^ The mechanism of VC is completely different from that of atherosclerosis, but the prediction of cardiovascular events is similar.^1^, ^4^, ^5^ At present, there are many research on atherosclerosis, and early treatment has become systematic, while early screening and treatment for VC are not available.^1^, ^6^

Early detection of VC is difficult, because a certain amount of calcification can be detected on CT.^12^, ^14^ The prediction model of VC is of great significance and plays a suggestive role, which is conducive to early detection of VC, especially in remote mountainous areas without good medical conditions.

At present, the gold standard for the evaluation methods of VC is the Agatston integral method, which mainly relies on the quantitative index of Coronary artery calcification CACS and is widely used to evaluate the volume of coronary artery calcification. However, this method is based on the examination of coronary computed tomography angiography, which costs a lot and requires intravascular injection of contrast agent, which may aggravate vascular injury or even endanger life safety and is not conducive to early clinical screening and evaluation.^26^ Chest CT scan can evaluate the density and extent of calcified plaques in the great arteries without the above adverse reactions.^12^, ^14^, ^15^, ^16^ However, in traditional methods, imaging reporting and diagnosis of VC by radiologists are time‐consuming and easy to miss, and the total social cost of evaluating VC is high.^3^ In addition, because only a certain amount of calcification can be detected by CT, early detection of VC is difficult.^14^, ^15^, ^16^ ML has been widely used in clinical risk prediction model recently due to its huge computing power and advantages of continuous autonomous learning.^17^, ^19^, ^20^, ^21^ The risk prediction model of VC based on chest CT scan is one of the main development directions to accurately assess the risk of VC. Previous studies have found that chest CT based on deep learning algorithm can automatically predict CACS scores, and this algorithm has shown good results in image deep learning tasks.^22^, ^23^ However, there are few studies on the establishment of VC risk prediction model based on ML algorithm and general demographic characteristics and clinical characteristics, and there is no prediction model of VC in Chinese type 2 diabetes population. At present, the research on VC risk prediction models in the world is still lagging behind, and the VC risk prediction model based on Chinese patients with type 2 diabetes has not yet been formed. This study is the first to apply ML approaches to VC risk prediction in Chinese patients with type 2 diabetes, and the model includes personal risk factors to identify high‐risk patients.

Based on the LR model, the Nomogram was made in this study, which has good predictive value and has been well applied in clinical practice. The AUC value of 0.749 (95%CI: 0.674−0.812), a specificity of 0.821 (95% CI: 0.775–0.866), and an accuracy of 0.784 (95% CI: 0.741–0.827). LR is a classification learning algorithm used to solve the problem of linear indivisibility of data sets. Its advantage is first model method is relatively simple, easy to implement. Second, the results can also clearly know the prediction probability of each category, not only the category of the training set can be predicted, but also the near‐like probability of the sample points of the prediction results can be obtained, and the probability of whether each sample point occurs can be calculated, which is usually used for the classification of diseases.^24^ Based on this feature of the LR model, this study established a nomogram to assess the individual risk of VC in type 2 diabetes patients at any time. It is very easy to use and to realize LR visualization. In clinical application, patients or physicians can input individual clinical characteristics into the nomogram at any time and anywhere, and the sum of individual scores of these 10 predictive variables can be obtained on the probability line to obtain the corresponding scale, namely the probability of occurrence of VC, so as to evaluate the risk of VC in patients with type 2 diabetes. There are several limitations to this study. First, this was a cross‐sectional study at a single center. Based on this, we plan to collect the follow‐up data of patients for 10 years after diagnosis and conduct a retrospective study to further verify the predictive ability of the model for VC risk, as well as the predictive efficacy of the model in different stages of type 2 diabetes, so as to better verify the generalization ability of the proposed model. Besides that we will conduct model optimization, external validation, and comparative experiments with larger sample sizes in the future.

## 5 CONCLUSIONS

This study is the first study to build a prediction model for VC based on ML algorithms in Chinese patients with type 2 diabetes. Using electronic medical records data, the prediction models based on eight ML algorithms were obtained: *k*‐NN, LightGBM, LR, MLP, naive bayes classifier, RF, SVM, and XGB models. The prediction performance of the model was evaluated by AUC, sensitivity, specificity, accuracy, and other indicators: NBC model has the best discrimination for predicting the risk of VC, and MLP model has good negative predictive value. Based on LR model, the nomogram can be constructed to visualize the results, which is more convenient and practical for clinical practice. In addition, the 10 important features screened by LASSO regression and their ranking are reliable: age, weight, fibrinogen, smoking history, body mass index, pulse pressure, diastolic pressure, total blood calcium, serum uric acid, and fasting blood glucose. Among them, serum uric acid as an important predictive feature of VC has attracted our attention in recent years.

## AUTHOR CONTRIBUTIONS

**Xue Liang**: Data curation; formal analysis; investigation; methodology; visualization; writing—original; writing—review and editing. **Xinyu Li**: Conceptualization; funding acquisition; resources; and supervision. **Guosheng Li**: Project administration; writing—review and editing. **Bing Wang**: Project administration; writing—review and editing. **Yudan Liu**: Writing—review and editing. **Dongli Sun**: Data curation. **Li Liu**: Data curation. **Ran Zhang**: Data curation. **Shukun Ji**: Data curation. **Wanying Yan**: Formal analysis; methodology; software. **Ruize Yu**: Formal analysis; methodology; software. **Zhengnan Gao**: Funding acquisition; project administration; supervision; writing—review and editing. **Xuhan Liu**: conceptualization; funding acquisition; methodology; resources; supervision. All authors have read and agreed to the published version of the manuscript.

## CONFLICT OF INTEREST STATEMENT

The authors declare no conflict of interest.

## Supporting information

Supporting information.

Supporting information.

Supporting information.

Supporting information.

## Data Availability

The data sets used and/or analyzed during the current study are available from the corresponding author on reasonable request.
