# Supplementary material for: A machine learning approach to predicting vascular calcification risk of type 2 diabetes: A retrospective study
Source: Clin Cardiol. 2024 Apr 2;47(4):e24264. doi: 10.1002/clc.24264 (PMC10985945; doi:10.1002/clc.24264)
Supplement: Supplementary file 1 — Supporting information. [file CLC-47-e24264-s001.pdf]

Supplementary Material

Supplementary Figures and Tables

Table S1. Candidate predictor variables.

Attribute number	Attribute name	Attribute description	Attribute type and measurement
1	Age	Age of patients	Numeric(years)
2	Sex	Sex of patients	Categorical<br>1 = Female; 0 =Male
3	Nation	Nation of patients	Categorical<br>1 = ethnic Han;<br>0 =not Han
4	Therapeutic Drugs		Categorical
		Use Statins / Fibrates or not	1 = use Statins / Fibrates;
		Use Oral antidiabetic agents or not	2 = use OADs;
		Use Insulin or not	3 = use Insulin;
		Use Calcium Calcium Entry Blockers or not	4 = use CCB;
		Use ARB or not	5 = use ARB;
		Use β-blocker or not	6 = use β-blocker;
		Use Diuretic or not	7= use Diuretic;
		Use ACEI or not	8 = use ACEI;
		Use Thyroid agents or not	9= use Thyroid agents;
		Use other related drugs or not	10= use other related drugs.

Attribute number	Attribute name	Attribute description	Attribute type and measurement
5	Hypertension	Whether patients have Hypertension	Categorical<br>1 = have hypertension;<br>0 = not have hypertension
6	CHD	Whether patients have coronary heart disease	Categorical<br>1 = have CHD;<br>0 = not have CHD
7	Smoking status	Smoking status of patients	Categorical<br>0 = never-smokers;<br>1 = current smokers;<br>2=ex-smokers
8	Drinking status	Drinking status of patients	Categorical<br>0 = never-drinkers;<br>1 = current drinkers;<br>2= ex-smokers
9	Family history	Whether patients have family history of disease	Categorical<br>1 = have family history of disease;<br>0 = not have family history of disease
10	Height	Height of patients	Numeric(cm)
11	Weight	Weight of patients	Numeric(kg)
12	BMI	Body mass index of patients	Numeric(kg/m^2)

Attribute number	Attribute name	Attribute description	Attribute type and measurement
13	HR	Heart rate of patients at admission	Numeric(beats per min)
14	DBP	Diastolic blood pressure of patients at admission	Numeric(mmHg)
15	SBP	Systolic blood pressure of patients at admission	Numeric(mmHg)
16	PP	Pulse pressure of patients at admission	Numeric(mmHg)
17	UPRO	Whether urine protein of patients is positive	Categorical<br>0 = "-" ;<br>1 = "+-" ;<br>2 = "+" ;<br>3 = "++" ;<br>4 = "+++" ;<br>5= "++++" or more
18	Scr	Serum creatinine level of patients	Numeric (umol/L)
19	BUN	Blood urea nitrogen level of patients	Numeric (mmol/L)
20	SUA	Serum uric acid level of patients	Numeric(umol/L)
21	tCa	serum total calcium level of patients	Numeric(mmol/l)
22	FIB	Fibrinogen level of patients	Numeric(g/l)
23	FPG	Fasting plasma glucose of patients	Numeric(mmol/L)

Abbreviations: ARBs = angiotensin receptor blockers; ACEIs = Angiotensin-Converting Enzyme Inhibitors.

Table S2. Best model parameters.

Models	Params
LightGBM	{'max_depth': 7, 'min_data_in_leaf': 80, 'num_leaves': 31, 'reg_alpha': 0.1}
KNN	{'metric': 'manhattan', 'n_neighbors': 13, 'weights': 'distance'}
XGBoost	{'gamma': 2, 'max_depth': 4, 'min_child_weight': 5, 'scale_pos_weight': 1, 'subsample': 0.6}
RF	{'max_depth': 50, 'max_features': 0.5, 'min_samples_leaf': 1, 'min_samples_split': 2, 'n_estimators': 500}
LR	{'C': 0.08858667904100823, 'penalty': 'l2'}
SVM	{'C': 10, 'gamma': 1, 'kernel': 'rbf'}
NB	{'var_smoothing': 0.3359818286283782}
MLP	{'hidden_layer_sizes': 12, 'max_iter': 200}

Abbreviations: k-NN = k-nearest neighbor; LightGBM = light gradient boosting machine; LR = Logistic regression; MLP = multilayer perceptron; NB = Naive Bayes; RF = Random forest; SVM = Support vector machine; XGB =XGBoost.

Table S3. Baseline characteristics of study population.

Characteristic	Training set N=1407	Test set N=352	*P Value**
Sex (n,(%))			0.431
Male	736 (52.3%)	193 (54.8%)	
Female	671 (47.7%)	159 (45.2%)	
Nation (n,(%))			0.628
ethnic Han	1379 (98.0%)	347 (98.6%)	
Non-ethnic Han	28 (1.99%)	5 (1.42%)	
Age (Mean±SD)	64.7±11.0	65.0±11.2	0.579
Height (Mean±SD)	167 ± 6.02	167 ± 5.72	0.641
Weight (Mean±SD)	71.55±9.13	70.47±11.86	0.062
BMI (Mean±SD)	25.5±2.82	25.1±3.84	0.106

Characteristic	Training set<br>N=1407	Test set<br>N=352	*P Value**
Smoking history (n,(%))			0.547
yes	320 (22.7%)	86 (24.4%)	
no	1087 (77.3%)	266 (75.6%)	
Drinking history (n,(%))			0.536
yes	230 (16.3%)	63 (17.9%)	
no	1177 (83.7%)	289 (82.1%)	
Family history (n,(%))			0.983
yes	207 (14.7%)	51 (14.5%)	
no	1200 (85.3%)	301 (85.5%)	
Hypertension (n,(%))			0.598
yes	787 (55.9%)	203 (57.7%)	
no	620 (44.1%)	149 (42.3%)	
coronary heart disease (n,(%))			0.191
yes	158 (11.2%)	49 (13.9%)	
no	1249 (88.8%)	303 (86.1%)	
Heart rate (Mean±SD)	78.9 ±8.81	79.0 ±9.60	0.840
Systolic blood pressure (Mean±SD)	143 ±21.5	142 ±22.2	0.496
Diastolic blood pressure (Mean±SD)	83.1 ±10.0	83.2 ±10.9	0.822
pulse pressure (Mean±SD)	57.1 ±17.7	55.9 ±16.6	0.259
Serum uric acid (Mean±SD)	304 ±82.8	302 ±80.5	0.653
serum total calcium (Mean±SD)	2.30 ±0.11	2.30 ±0.12	0.648
Fibrinogen (Mean±SD)	3.61 ±1.15	3.76 ±1.25	0.047
Fasting plasma glucose (Mean±SD)	8.91 ±3.19	8.91 ±3.40	0.990
Serum creatinine level (Mean±SD)	67.3 ±33.6	67.3 ±36.6	0.987
Blood urea nitrogen level (Mean±SD)	6.06 ±2.09	6.00 ±1.85	0.590

Characteristic	Training set<br>N=1407	Test set<br>N=352	*P Value**
urine protein (n,(%))			0.309
"-"	1224 (87.0%)	307 (87.2%)	
"+-"	21 (1.49%)	9 (2.56%)	
"+"	108 (7.68%)	23 (6.53%)	
"++"	43 (3.06%)	9 (2.56%)	
"+++"	11 (0.78%)	3 (0.85%)	
"++++ or more"	0 (0.00%)	1 (0.28%)	
Therapeutic Drugs (n,(%))			0.568
Statins / Fibrates	15 (1.07%)	6 (1.70%)	
Oral anti-diabetic agents	44 (3.13%)	14 (3.98%)	
Insulin	69 (4.90%)	12 (3.41%)	
Calcium Calcium Entry Blockers	42 (2.99%)	12 (3.41%)	
ARBs	9 (0.64%)	0 (0.00%)	
β-blocker	5 (0.36%)	1 (0.28%)	
Diuretics	1 (0.07%)	1 (0.28%)	
ACEIs	6 (0.43%)	1 (0.28%)	
Thyroid agents	3 (0.21%)	0 (0.00%)	
Others	1213 (86.2%)	305 (86.6%)	

Values were presented as mean ± standard deviation or number (column percent) as appropriate.

*P value is obtained from a t-test for continuous variable and a Chi-square test for a categorical variable. $P < 0.05$ was considered statistically significant.

Abbreviations: ARBs = angiotensin receptor blockers; ACEIs = Angiotensin-Converting Enzyme Inhibitors.

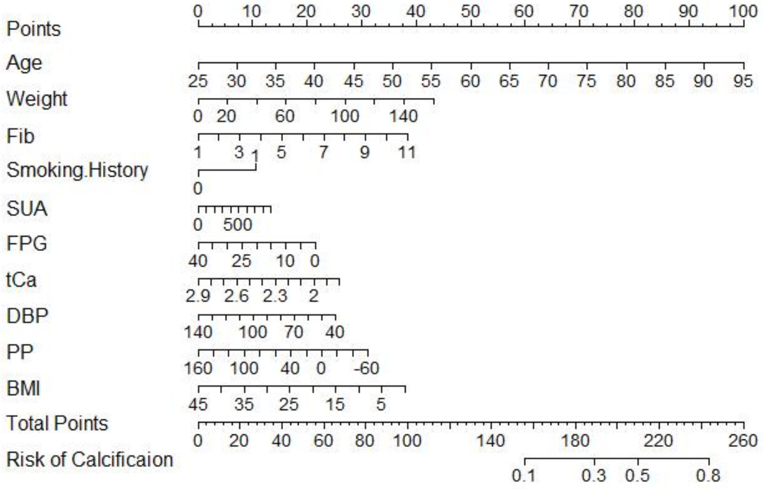

Figure S1. Nomogram with Lasso selected features for predicting VC risk of patients with T2DM.

To estimate the probability of VC, mark patient values at each axis, draw a straight line perpendicular to the point axis, and sum the points for all variables. Next, mark the sum on the total point axis and draw a straight line perpendicular to the probability axis.

Abbreviations: BMI = Body mass index; DBP = Diastolic blood pressure; PP = Pulse pressure; SUA = Serum uric acid; FPG = Fasting plasma glucose; tCa = serum total calcium; FIB = Fibrinogen.

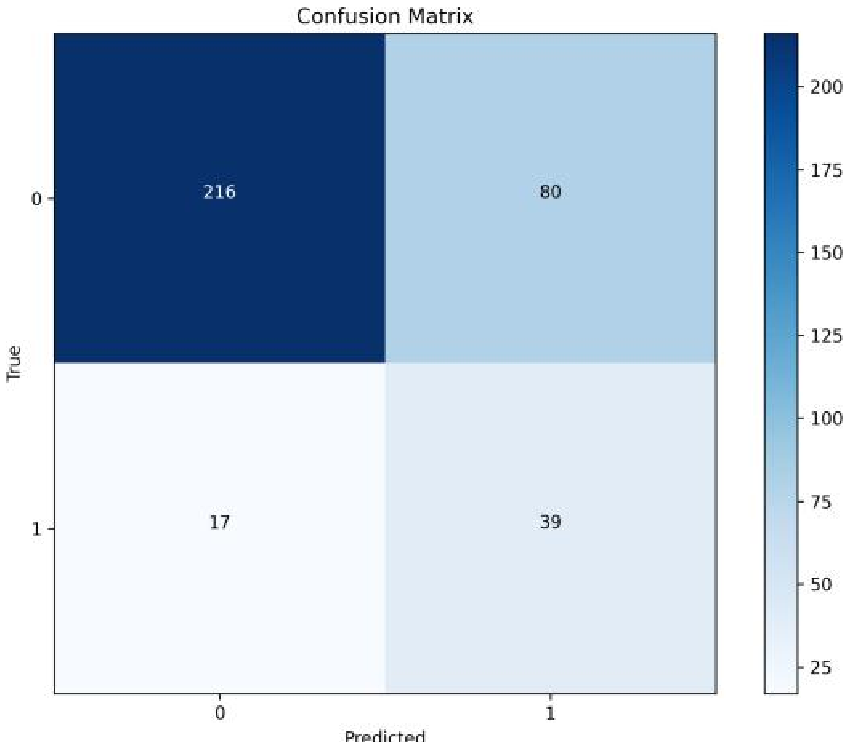

Figure S2. Confusion Matrix of the NB model and common performance metrics calculated from it.

The TP (true positives) were 216; FP (false positives) were 80; FN (false negatives) were 17; TN (true negatives) were 39. We calculated the rate of TP, FP, FN, and TN. Also, we calculated the precision, accuracy, etc.
